# The Importance of Early Diagnosis and Treatment in Septic Arthritis of the Temporomandibular Joint: A Case Report

**DOI:** 10.7759/cureus.64480

**Published:** 2024-07-13

**Authors:** João Oliveira, Margarida Mesquita, Sofia Correia, Margarida Colino

**Affiliations:** 1 Maxillofacial Surgery, Unidade Local de Saúde de Coimbra, Coimbra, PRT

**Keywords:** temporomandibular disorders, temporomandibular joint, infectious arthritis, septic arthritis, infection

## Abstract

Septic arthritis of the temporomandibular joint (SATMJ), primarily caused by bacterial infections, is a rare condition with a diverse etiology that is inadequately documented in the literature, resulting in the absence of standardized treatment protocols. Its nonspecific clinical presentation often leads to misdiagnosis as other temporomandibular disorders, delaying diagnosis and treatment and potentially causing severe complications in the absence of established therapeutic guidelines.

The main objective of this article is to report a case of a 61-year-old female with diabetes who was undergoing prolonged corticosteroid therapy and presented with pain, swelling in the right pre-auricular area, and progressive limitation in mouth opening, with no history of facial trauma, where the early diagnosis and isolation of *Staphylococcus aureus *after a single-port arthrocentesis prompted the timely adjustment of the treatment regimen, significantly influencing the outcome by mitigating the risk of complications. Additionally, this report includes a comprehensive literature review, highlighting the crucial importance of this prompt intervention to achieve a favorable clinical outcome.

## Introduction

Septic arthritis of the temporomandibular joint (SATMJ) is an inflammatory condition primarily instigated by bacterial infection, commonly associated with organisms such as *Staphylococcus *spp., *Neisseria gonorrhoeae*, *Haemophilus influenzae*, and *Streptococcus *spp. [1,2], as well as fungi, viruses, or parasites, albeit less frequently [3]. Its etiology can be attributed to the hematogenous dissemination of pathogens from distant infection sites or the contiguous spread from adjacent sources (e.g., odontogenic, otologic, or upper respiratory tract infections) [1,4,5], with direct inoculation, often secondary to trauma or surgical procedures, also constituting a potential causative mechanism [2,4].

Given its relatively rare occurrence, SATMJ is inadequately documented in the scientific literature, thus lacking standardized treatment protocols founded on empirical evidence [1,2]; furthermore, its nonspecific clinical presentation, which overlaps with various temporomandibular joint (TMJ) disorders, complicates accurate diagnosis [1,5,6].

The untimely or inaccurate identification and management of SATMJ can precipitate substantial functional impairment and significant morbidity, with potential complications including joint ankylosis, condylar resorption, osteomyelitis, and intracranial abscess formation [2,5,6].

This article aims to present a case of SATMJ and conduct a comprehensive literature review, emphasizing the critical significance of prompt and appropriate diagnosis and treatment to ensure a favorable clinical outcome.

## Case presentation

A 61-year-old female diagnosed with pemphigus foliaceus and receiving a tapering regimen of prednisolone and azathioprine, currently at doses of 20 mg once daily and 50 mg twice daily, respectively, presented to the emergency department with a 15-day history of pain and swelling in the right pre-auricular area, accompanied by progressive limitation in mouth opening, without any impact on speech or swallowing and no systemic signs or symptoms. Her medical history included obesity, hypertension, diabetes mellitus, and a past episode of syphilis, which appeared to have been successfully treated.

Upon physical examination, the patient presented with painful swelling in the right pre-auricular area, which did not show fluctuation upon palpation. Palpation indicated difficulty in assessing right condylar excursion, suggesting trismus without deviation of the mandibular midline, in a patient who was completely edentulous in the upper dental arch and exhibited clear saliva drainage through Stenson's duct.

**Figure 1:**
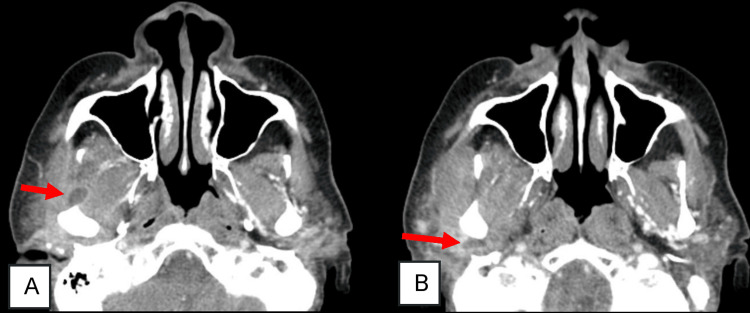
Computed tomography (CT) scan before treatment CT scan revealing two small abscess collections (red arrows) surrounding the right mandibular condyle, anteriorly (A) and posteriorly (B)

Laboratory analyses demonstrated an elevated C-reactive protein (CRP) and erythrocyte sedimentation rate (ESR) in the absence of leukocytosis. In addition to cellulitis and right parotid gland enlargement, a contrast-enhanced computed tomography (CT) scan of the maxillofacial region revealed the presence of two small abscesses encircling the right mandibular condyle, each measuring approximately 1 cm in diameter (Figure 1).

In light of suspicion regarding septic arthritis affecting the right temporomandibular joint without a discernible etiology, the patient underwent hospitalization for comprehensive diagnostic assessment, and empirical antibiotic therapy was initiated consisting of ceftriaxone and vancomycin. Blood cultures yielded negative results, while serological examinations indicated immunity to hepatitis B, prior infection with *Mycoplasma pneumoniae*, and seemingly resolved syphilis, with negative findings for HIV and hepatitis C.

Within 48 hours of initiating medical intervention, a single-port arthrocentesis procedure was performed under local anesthesia on the right temporomandibular joint (TMJ), involving needle insertion 10 mm forward from the midtragus and 2 mm inferior to the canthotragal line, aspirating approximately 2 cc of purulent fluid exclusively for microbiological (both aerobic and anaerobic) and mycobacterial culture due to limited sample availability, followed by a 100 mL lavage with lactated Ringer solution.

On the sixth day of hospitalization, *Staphylococcus aureus* isolated from aspirated fluid demonstrated sensitivity to clindamycin, vancomycin, oxacillin, gentamicin, and trimethoprim/sulfamethoxazole and resistance to penicillin G and erythromycin, prompting the adjustment of the treatment regimen to trimethoprim/sulfamethoxazole.

**Figure 2:**
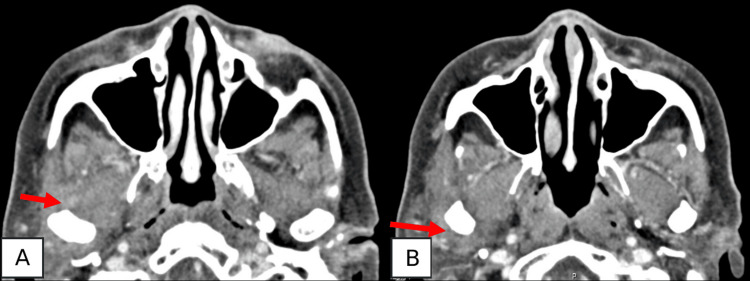
Computed tomography scan two weeks after arthrocentesis Complete resolution of the abscess collections (red arrows), anteriorly (A) and posteriorly (B)

On the 16th day of hospitalization, significant improvement in pain and increased mouth opening were observed, supported by the complete resolution of abscesses as confirmed by subsequent CT imaging (Figure 2).

As a result, the patient was discharged after 22 days with instructions to continue antibiotic therapy for an additional eight days and to perform jaw exercises until starting physical therapy, which was requested.

**Figure 3:**
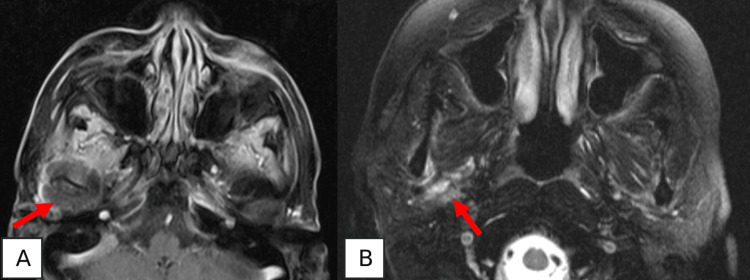
Magnetic resonance imaging four months after treatment No evidence of organized abscesses. (A) Axial T1 FS post-gadolinium image showing synovial thickening with slight enhancement (red arrow), suggestive of synovitis with some fibrosis. (B) Axial T2 FS image showing slight condylar edema (red arrow) FS: fat-saturated

A follow-up magnetic resonance imaging (MRI) conducted four months later revealed the anterior dislocation of the right articular disc, the disruption of the condylar cortex, synovitis with degenerative changes, and probable fibrotic alterations, with no evidence of organized abscesses (Figure 3).

Despite these findings, the patient reported pain relief and no functional impairments, and the option was made to continue with physical therapy, with no complications to date except for occasional pain resolved with conservative measures.

## Discussion

This article describes a case of early-diagnosed and promptly treated SATMJ, highlighting its favorable influence on the prognosis.

SATMJ is a rare and poorly understood clinical condition that should be considered in patients presenting with acute and severe pre-auricular pain and swelling, accompanied by trismus, mandibular deviation from the dental midline, and malocclusion [1-3], particularly when other causes of arthritis have been ruled out and there is no history of facial trauma [1,2,6]. SATMJ typically presents as a monoarticular condition, though the case described here involves a middle-aged female patient, contrasting with its more typical manifestation in young males [2,3], and may include systemic symptoms such as fever and general malaise [2,3].

In most cases, including the one we presented here, hematogenous spread from a distant infection site remains the primary causative factor, often difficult to detect [1-4], with the TMJ being particularly vulnerable to this route of transmission due to its highly vascularized synovial membrane and the absence of a basement membrane [6,7]. Other documented etiologies include contiguous spread from adjacent infections, such as those arising from odontogenic, otologic, parotid, or upper respiratory tract sources [1,2,5], along with less frequent occurrences of direct inoculation from trauma or surgical interventions [2,3,5].

The patients at increased risk include those with systemic and autoimmune conditions (such as diabetes and rheumatoid arthritis), individuals undergoing prolonged systemic corticosteroid therapy, immunocompromised individuals, those with sexually transmitted diseases (STDs) [6-8], and those with previous joint alterations from trauma, joint injuries/diseases, or treatment with bisphosphonates [2,5-9]. In the specific case described herein, aside from diabetes, the patient was undergoing prolonged corticosteroid therapy for pemphigus foliaceus, with STDs ruled out.

Early diagnosis is crucial for achieving a favorable prognosis and reducing the risk of significant intra-articular structural damage [3,4], relying primarily on clinical assessment supported by imaging modalities, laboratory investigations, and synovial fluid analysis [2-4].

Leukocyte levels and inflammatory markers such as CRP and ESR are often elevated [2,3], with CRP levels providing reliable indicators of severity and aiding in confirming resolution [2]. However, the diagnostic utility of serum leukocyte levels is debatable since they may remain within normal ranges [2,4,7,8]. Although negative, blood cultures are useful for diagnosis, especially when it is unlikely to obtain intra-articular material [3,8].

Given the broad spectrum of potential differential diagnoses, imaging modalities serve as indispensable adjunct examinations [3-5,7]. Panoramic radiographs provide rapid and straightforward assessments for detecting significant osseous joint alterations and increased joint space due to pus and inflammatory exudate accumulation [1,5], although they pose a higher risk of false-negative results, especially in the early stages of infection [2,5]. Consequently, many researchers advocate for CT and MRI as preferred and reliable diagnostic tools, with CT being valuable for the early detection of bone changes and abscess collections and MRI facilitating earlier and more precise identification of joint effusions while offering superior resolution of joint surfaces [1-5,8,9].

Synovial fluid collection is crucial for confirming the diagnosis of septic arthritis [1,3,4], with surgical drainage methods including arthrocentesis, arthroscopy, and arthrotomy [1,4,7]. Arthroscopy allows the direct visualization of intra-articular structures, aiding precise diagnosis and targeted therapy, but is typically reserved for severe cases due to its complexity [1,6-8]. Therefore, arthrocentesis is commonly employed for drainage, enabling material collection for Gram stain, culture, and sensitivity analyses, as well as joint lavage [1,5]. The evaluation of aspirated fluid color and turbidity serves as infection indicators [1,3-5,8], while surgical joint drainage supports cleansing and may involve multiple aspirations to expedite healing [10]. In cases of significant joint damage, condylar shaving or condylectomy may be necessary [10].

The predominant microorganism frequently identified is *Staphylococcus aureus*, with reported cases also involving *Staphylococcus saprophyticus*, *Streptococcus pyogenes*, *Neisseria gonorrhoeae*, and *Haemophilus influenzae* [1,2,6], while fungal, viral, or parasitic joint infections are less common but possible [3]. Nevertheless, in numerous instances, synovial fluid culture results prove negative, attributed not only to the frequently limited quantity of collected material but also to the initiation of empirical antibiotic therapy by most patients prior to aspiration [1,2,6].

In the presented case, the suspicion of septic arthritis arose from clinical and imaging assessments, followed by arthrocentesis to confirm the diagnosis and perform joint lavage, with *Staphylococcus aureus* isolated as the predominant pathogen in this condition.

In SATMJ, while surgical joint lavage is essential for treatment, the prompt initiation of antibiotic therapy is fundamental [1,5,7,8], aiming to prevent complications such as irreversible joint destruction, intracranial abscesses, ankylosis, and condylar osteomyelitis [1,3]. Initially, broad-spectrum parenteral and empirical antibiotics, such as third-generation cephalosporins, are administered [1,2]. In settings with a high prevalence of methicillin-resistant *Staphylococcus aureus* (MRSA), vancomycin or sulfonamides may be indicated [6]. Subsequently, upon the receipt of culture and sensitivity results, a more tailored therapeutic approach is adopted to enhance efficacy and potentially shorten treatment duration [1,2,10], with antibiotic therapy typically exceeding 30 days, contingent on clinical response [1,4,6].

The therapeutic regimen administered to this patient adheres to literature recommendations, starting with parenteral broad-spectrum antibiotic therapy comprising ceftriaxone and vancomycin for six days. Following sensitivity test results, treatment was switched to trimethoprim/sulfamethoxazole for an additional 24 days, with the final eight days administered orally.

Early physical therapy, including controlled jaw exercises, is crucial to improve mouth opening range after the acute infection phase [1,3,8] and is pivotal in preventing complications such as fibrosis and ankylosis [3,7], a practice that was advocated for this patient.

Following the resolution of the acute event, the majority of patients typically regain full TMJ function; however, there is a risk of infection recurrence and subsequent sequelae such as chronic pain or restricted mouth opening due to fibrosis or ankylosis, which may manifest more than one year after the initial episode, demanding long-term follow-up [1,2,4,8,10].

In this specific case, the presence of osteoarthritis signs within the affected joint suggests that age-related factors may not be the sole cause; however, due to the absence of functional impairments in the patient, a conservative treatment approach has been chosen, and to date, no further complications have been reported.

## Conclusions

We presented a case of septic arthritis involving the temporomandibular joint in a middle-aged female patient, a rare occurrence documented infrequently in the literature. Given its nonspecific clinical presentation, it can mimic other temporomandibular disorders, highlighting the necessity of a comprehensive diagnostic approach comprising a detailed clinical history, thorough examination, imaging studies, complementary tests, and synovial fluid cultures to achieve an accurate diagnosis.

Although definitive treatment guidelines are lacking, early identification and management, involving the administration of broad-spectrum antibiotics and surgical drainage, are vital to prevent complications and mitigate long-term sequelae, as evidenced in this case. Nonetheless, diligent long-term follow-up care remains indispensable.
